# Online spike-based recognition of digits with ultrafast microlaser neurons

**DOI:** 10.3389/fncom.2023.1164472

**Published:** 2023-07-03

**Authors:** Amir Masominia, Laurie E. Calvet, Simon Thorpe, Sylvain Barbay

**Affiliations:** ^1^Université Paris-Saclay, CNRS, Centre de Nanosciences et de Nanotechnologies, Palaiseau, France; ^2^LPICM, CNRS-Ecole Polytechnique, Palaiseau, France; ^3^CERCO UMR5549, CNRS—Université Toulouse III, Toulouse, France

**Keywords:** photonic hardware, temporal coding, rank-order code, spiking neurons, microlasers, receptive fields

## Abstract

Classification and recognition tasks performed on photonic hardware-based neural networks often require at least one offline computational step, such as in the increasingly popular reservoir computing paradigm. Removing this offline step can significantly improve the response time and energy efficiency of such systems. We present numerical simulations of different algorithms that utilize ultrafast photonic spiking neurons as receptive fields to allow for image recognition without an offline computing step. In particular, we discuss the merits of event, spike-time and rank-order based algorithms adapted to this system. These techniques have the potential to significantly improve the efficiency and effectiveness of optical classification systems, minimizing the number of spiking nodes required for a given task and leveraging the parallelism offered by photonic hardware.

## 1. Introduction

Photonic artificial neural networks can open great prospects for the realization of fast and energy efficient image recognition tasks. The advantages of photonic systems include integration, very small dissipation during information transport, ultra-fast response times (sub nanosecond) and, due to the number of controllable nonlinearities in optical materials, a wealth of physical properties useful for information processing. In particular, spiking or excitable photonic systems (Nahmias et al., 2013; Feldmann et al., 2019; Skalli et al., 2022) are exceptional candidates for building third generation neural networks, which are predicted to signficantly improve power consumption and augment computational capabilities (Maass, 1997; Thorpe et al., 2001; Stöckl and Maass, 2021).

Recent research has generated great interest in the use of physical reservoir computing, where the complex dynamics of a physical system are exploited to project input data into a larger dimensional space. Its output is then typically classified in an offline computation using a relatively simple method like a ridge regression (Tanaka et al., 2019; Nakajima, 2020). Such methods include a wide variety of different systems such as nano-electronic spin-torque nanoscillators (Torrejon et al., 2017), organic electrochemical networks (Cucchi et al., 2021), and photonics (Lugnan et al., 2020) using, e.g., optoelectronic oscillators (Larger et al., 2017) or spiking vertical cavity surface emitting lasers (Robertson et al., 2022; Owen-Newns et al., 2023). One exception to resorting to a software based step involved all-optical time-series prediction using passive devices (Bueno et al., 2018) and emulated spiking-based recognition of four different letters in the optical domain (Feldmann et al., 2019). Removing this offline step can significantly improve the response time and energy efficiency of these systems. In this study, we present an algorithmic approach to image recognition that utilizes ultrafast photonic spiking neurons, allowing for a recognition by solely observing the responses of these neurons. This work represents an important step toward the development of more efficient and effective methods for image classification.

**Figure 1:**
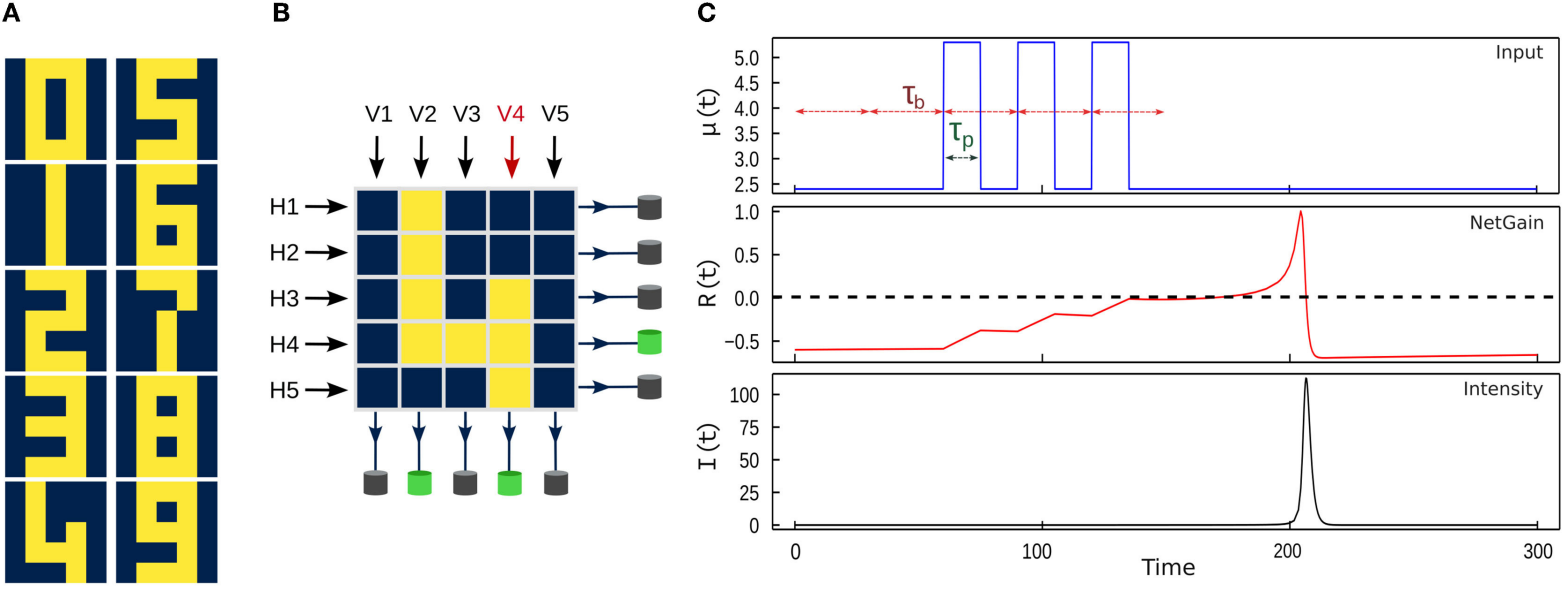
**(A)** The 10 digits used in this study. Digits consist of 5 × 5 binary pixels in each image. **(B)** Horizontal and vertical receptive fields define input bit sequences, which are fed into the neuron through the time varying pump μ_1_(*t*). Depending on the neurons internal parameters, the neurons can fire or not. **(C)** Illustration of the neuron response to the *V*_4_ receptive field with the input bit sequence [0, 0, 1, 1, 1]. (Top): Corresponding input pump μ_1_(*t*) (Equation 2) with bit duration τ_*b*_, pump pulse duration τ_*p*_ and pump amplitude *c*. (Middle): Net gain of the micropillar *R*(*t*). (Bottom) Output intensity *I*(*t*).

We investigate a model task of digit classification utilizing simplified 5 × 5 binary pixel images of the 10 digits, as depicted in Figure 1A. The photonic nodes used here are semiconductor-based micropillar lasers with integrated saturable absorber. Our technique is based on tuning the key physical parameters of photonics microlaser neurons, such as the bias pump, input bit time and intensity, so that their response consists of a single spike that is sensitive to certain features of the data. The optical spiking neurons exhibit the fundamental properties of biological neurons, such as excitability, relaxation, and a refractory period, but are six orders of magnitude faster (Pammi et al., 2020).

The inspiration for this technique is found in the biological concept of a receptive field (RF), where neurons exhibit a selectivity to only certain stimuli, resulting in a very sparse and therefore energy efficient encoding of information. Our method is also related to temporal encoding schemes known as time to first spike (Bonilla et al., 2022), which are gaining increasing interest due to its low energy consumption while maintaining an excellent computational performance (Abderrahmane et al., 2020; Kheradpisheh and Masquelier, 2020; Park et al., 2020; Gardner and Grüning, 2021; Guo et al., 2021). Such encoding is believed to play a crucial role in several cognitive processes, such as memory retention and decision-making. It is particularly well-suited for applications where speed is required. While we explore here a simple classification task, our approach may also be used for ultra-fast feature selection prior to a larger classification task, which will benefit the system by reducing its dimensionality. What makes our results unique in the literature is not just the consideration of different temporal schemes based on an optically spiking neuron, but the use of a more complex neuron model for hardware compared to the integrate and fire neurons previously explored.

## 2. Methods

### 2.1. Model for spiking microlaser neuron

The model we use to describe the microlaser neurons derives from the Yamada (1993) model, which has proven to adequately predict the response of such systems in various configurations (Barbay et al., 2011; Terrien et al., 2020). It consists of three dimensionless coupled ordinary differential equations:


(1) $$\begin{array}{l} \dot{I}=I\left(G-Q-1\right)+\beta (G+\eta )2\\ \dot{G}=\gamma_{G}\left(\mu_{1}(t)-G(1+I)\right)\\ \dot{Q}=\gamma_{Q}\left(\mu_{2}-Q(1+sI)\right) \end{array}$$

where *I* is the intracavity intensity, *G* is the gain, *Q* is the saturable absorption, and γ_*G*_ and γ_*Q*_ are the gain and the SA relaxation rates, respectively. All the parameters are scaled to the cavity photon lifetime, which is on the order of 1–2 ps in physical units. The parameter β models the amout of spontaneous emission coupled to the laser cavity mode. We define the important quantity, net gain, as *R* = *G*−*Q*−1. The microlaser is pumped with a strength μ_1_, which can be adapted to either electrical or optical pumps, and μ_2_ is the linear unsaturated absorption. The saturation parameter is *s*, which, for semiconductor materials here, takes the value *s*≃10. We use typical parameters for semiconductor materials, such that γ_*G*_ = γ_*Q*_ = 0.005, μ_2_ = 2, η = 1.4, and β = 10^−4^. Note that the gain and SA recombination rates are small (γ_*G, Q*_≪1), resulting in a slow-fast nonlinear system. The intensity dynamics are essentially governed by the net gain (at least in the linear regime): if *R*>0 laser intensity increases, and if *R* < 0 it decreases. The nonlinear terms in the gain and SA equations describe stimulated emission and absorption processes, leading either to light amplification or (saturable) absorption. The main physically controllable parameter is the pump μ_1_(*t*).

These equations have been studied theoretically in Dubbeldam and Krauskopf (1999) in the limit β → 0. When the pump μ_1_ is increased from 0, the laser starts in its off state and no light is emitted until the laser threshold is reached. Above the laser threshold (μ_1_>μ_1, *th*_ = 1+μ_2_), the microlaser is in the self-pulsing regime and emits a train of short pulses. Just below the laser threshold, the microlaser is in the excitable regime: it has a stable quiet state corresponding to no laser emission; if it is perturbed above a certain threshold (the excitable threshold), it emits a fast calibrated pulse in response and returns back to its quiet state in a time corresponding to the absolute and relative refractory periods. Experimentally, the pulse duration is about 200 ps and the relative refractory period is of the order of a few hundred piocseconds, typically 350 ps or more (Selmi et al., 2014). The spiking microlaser also displays spike latency (Selmi et al., 2016) and temporal summation (Selmi et al., 2015). It thus has all the main ingredients of a biological neuron from a computational point of view.

In this study, we focus on the excitable regime, where the microlaser can be considered as a photonic spiking neuron. Input signals can act on *I* or *G*, and are defined, respectively as coherent or incoherent. We consider the latter case, which is more practical experimentally, and input the information in the system on the pump parameter μ_1_(*t*), with a coding scheme that will be explained below.

### 2.2. Input coding

The data to be classified consists of images of digits, each of which is made up of 25 binary pixels (Figure 1A). These images are input into the micropillar by translating the value of each pixel in a row (*H*_*i*_) or column (*V*_*i*_) into a corresponding time varying pump value relative to the base pump value (μ_0_). This process is illustrated in Figure 1B). The input pump coding is defined by each input bit *p* from the given receptive field (*H*_*i*_ or *V*_*i*_) such that:


(2) $$\begin{array}{l} \mu_{1}\left(t\right)=\mu_{0}+\underset{i}{\sum }cp_{i}{\text{Π}}_{\tau_{p}}\left(t-i\tau_{b}\right) \end{array}$$

where Π_τ_*p*__(*t*) is a boxcar function of duration τ_*p*_. The time varying pump value is calculated as the sum of the base pump value μ_0_ and of the translated bit sequence, each bit having duration τ_*b*_≥τ_*p*_ and the added pump amplitude corresponding to the bit “1” being *c* (0 otherwise). The control parameters τ_*b*_ and τ_*p*_ are determined by physical considerations. If τ_*b*_ is much larger than the relative refractory period of the microlaser, the input bits will not interact since the system can reach steady state after each input pump pulse. By choosing the bit time τ_*b*_ slightly less than the relative refractory time, consecutive bits can be summed up due to temporal summation and the system can be made to fire only after a certain sequence is met. This is illustrated in Figure 1B) where the three consecutive “1” part of the input bit sequence “00111” allows the net gain *R*(*t*) to increase slightly above zero and thus elicit a spike in the laser intensity. Note that in this case, the system only fires after at least three consecutive input “1”s. The pump pulse width τ_*p*_ is chosen in conjunction with the pump amplitude *c* since, in the small pump pulse duration limit, the physically relevant quantity for the system is the pump pulse energy, i.e., the product τ_*p*_*c*.

## 3. Results

### 3.1. Horizontal and vertical receptive fields

Our approach to digit recognition is based on the temporal summation property of neurons (Koch, 2004), which results in receptive fields for vertical and horizontal features in the input image. Temporal summation, a.k.a. integration, occurs when the neuron integrates the individual stimuli into a single, stronger signal. It has been shown to be important biologically for numerous cognitive functions such as sensing pain (Price et al., 1977), seeing in dim light (Warrant, 1999), and auditory perception (Heil and Neubauer, 2003). It has been demonstrated in a photonic spiking neuron in Selmi et al. (2015) and used for ultrafast image processing using VCSELs in Hejda et al. (2022) and Robertson et al. (2022).

As shown in Figure 1, the majority of the features of each digit are oriented horizontally and vertically, and often consist of aligned blocks of pixels. This observation will drive our approach to the classification task. Our strategy is based on the manipulation of the physical parameters of the micropillar neurons such that they respond to specific input patterns. As shown in Figure 1, by encoding the pixels into pump values and sending them row by row or column by column to the corresponding micropillar neurons, we can elicit a response in the form of a spike if a certain pattern is detected (in this case, three perturbations in a row). This approach allows us to effectively classify the digits based on their distinctive features.

A salient feature observable in all the images in Figure 1 is the presence of either three or four perturbations arranged in a contiguous row or column. Furthermore, the digits 2 and 5 possess symmetrical features, requiring the implementation of neurons with new sets of parameters to detect the patterns “11101” or “10111.” By exploiting the principle of temporal summation, the parameters of the micropillar can be optimized such that the arrival of either of these perturbation patterns results in a net gain above the threshold (*R*≥0), leading to the generation of a spike. This can be used subsequently as an indicator of the detection of a specific pattern, making the classification in an experimental implementation relatively straightforward. Alternatively, the latency of the resultant spike can be utilized as a means of differentiating between the two primary patterns, as shown below.

### 3.2. Feature detection

**Figure 2:**
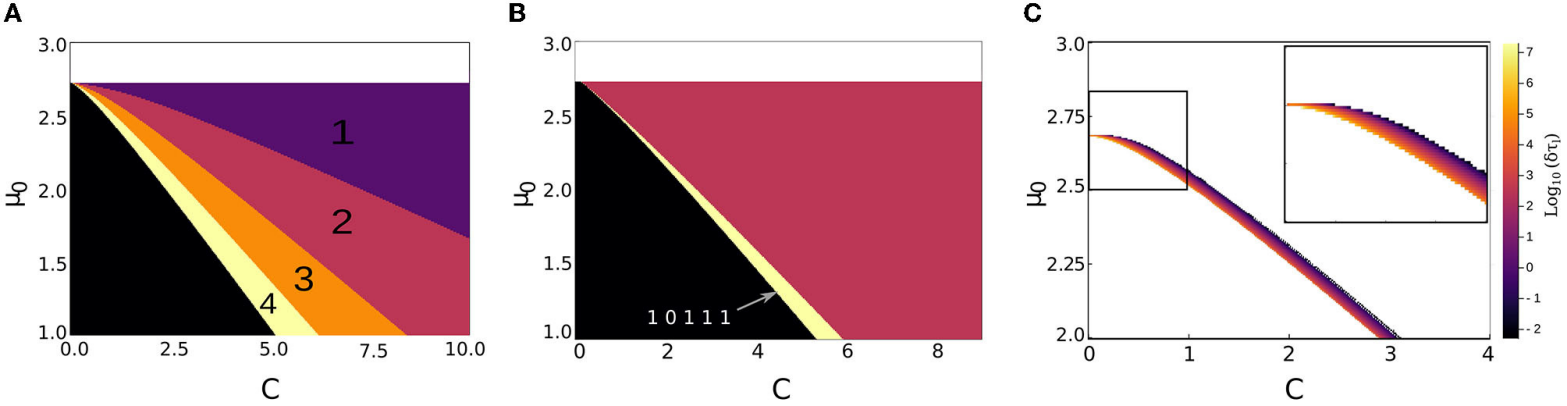
**(A)** Minimum number of consecutive “1” bits in the input sequence required to excite the photonic neuron. The region μ_0_ ⪆ 2.75 for the pump value corresponds to the self-pulsing regime and does not require any additional perturbations to produce a spike. **(B)** Parameter region where the input sequence 10111 is distinguishable. **(C)** Spike latency difference (in log_10_ scale) for sequences of three and four consecutive “1” bits, as a function of pump values and coefficients. The other parameters are τ_*b*_ = 50 and τ_*p*_ = 30.

We aim to investigate the parameter regime in which micropillars exhibit a spike response to a specific pattern while remaining insensitive to other patterns. To accomplish this objective, a comprehensive sweep of the relevant parameter space was conducted. The parameters that were varied in this sweep include the pump value (μ_1_), as well as the intensity of the perturbation, represented by the coefficient *c*. Furthermore, the effects of two additional parameters, namely the bit- and perturbation-times were also considered. The bit-time represents the duration of time allocated for each pixel to influence the system, while the perturbation-time denotes the duration of the perturbation when it is present. Through systematic variation of these parameters, we were able to determine the optimal parameter values that lead to the desired behavior in the micropillars. As demonstrated in Figures 2A, B, the distinction between various features is possible by a proper choice of the parameters (μ) and (*c*). Interestingly, the summation being a time dependant process, the input sequences “11101” and “10111” can be distinguished in a sizeable parameter range. Here, the bit-time and perturbation-time were fixed at 50 and 30 units of simulation time, respectively. These values serve to broaden the parameter regions for each pattern, thus enabling greater accuracy in identification.

### 3.3. Output coding with spike latency

Spike latency, also known as the temporal delay, is a nonlinear interval between the presentation of a stimulus to a neuron and the subsequent generation of an action potential as a response. This latency period is of paramount importance in neuron activation as it enables encoding the strength of the stimulus in the time domain (Fujii et al., 1996) a process known as temporal coding. In general, spike latency is a critical aspect of neuronal function and plays a pivotal role in a plethora of brain processes.

As an alternative to the previous coding scheme, we can take advantage of the fact that the temporal stamp for each spike, in the event of receiving any of the two predominant patterns, will be unique to that specific pattern. Through this approach, we can further diminish the feature space of the original dataset. In order to effectively implement this method experimentally, it is imperative to have a substantial disparity between the spike latencies of the two dominant patterns (three or four perturbations in a row). As depicted in Figure 2C, we conducted a comprehensive sweep over the same parameters, while maintaining the values of τ_*b*_ and τ_*p*_ as previously established. The spike latency diverges to infinity at the excitable threshold and decreases strongly when the excitation increases, until saturating to some non-zero value. This strong variation can be seen in Figure 2C where the spike latency difference varies over several orders of magnitude. It shows that a proper choice of parameters can allow for a large spike latency when needed to ease the feature distinction. In the vicinity of the excitable threshold, we can find the parameter region where the difference grows exponentially toward infinity, thus providing a window where the detection of two patterns will be eased. Indeed, from an experimental point of view, the system would also be subject to noise and large latencies would also be accompanied by large fluctuations of the latency time. Thus, a large latency difference can be useful to differentiate among input features.

### 3.4. Classification

We present three classification methods based on the encoding of the output spikes. One relies only on the presence or absence of a spike related to a specific feature (event based). The second incorporates the timing information of the spike arrival (spike-time based). The last one depends on the arrival order of the spikes (rank order based). As we will show, this choice can have a significant impact on the performance of the model and on the reduction of the overall computational cost. In the present classification methodology, the vertical and horizontal receptive fields (V and H, respectively) allow the retrieval of particular features at specific positions in the input image. This is rendered possible by fixing the pair of parameters, μ_0_ and *c*, for each considered feature.

#### 3.4.1. Event base coding

A time-independent classification approach is employed, relying on an event-driven coding strategy. In this method, each receptive field is input into a photonic neuron with different parameters μ_0_ and *c*, which are adjusted to detect the desired input feature corresponding to the presence or absence of a specific sequence, as calculated in Figure 2A. As shown in Table 1a, the three most significant sequences that result in a successful classification of the 10 digits are found to be “111,” “1111,” and “10111.” The parameters chosen to detect the sequences “111” and “1111” are μ_0_ = 1.25 and *c* = 6 and *c* = 5.5, respectively. In order to separate the classes corresponding to digits 2 and 5, it is necessary to introduce a feature to differentiate between the sequences “10111” and “11101.” Based on Figure 2B, we choose a feature with μ_0_ = 1.25 and *c* = 5.5. As a result, we show in Table 1a the codes associated to the different features tested. From these, it is clear that only 10 features are necessary to distinguish between the digits: {*H*1, *H*3, *H*5, *V*2, *V*4} associated to “111,” {*V*2, *V*3, *V*4} associated to “1111” and {*V*2} or {*V*4} associated to “10111.” We can eventually add {*V*3} tuned to detect “1111” if we exclude the empty code for digit “1.” This method can thus classify the digits using 10 neurons in parallel, provided one inputs the data of chosen receptive fields. The classification time taken will be on the order of 5τ_*b*_+τ_*l*_, τ_*l*_ being the largest latency time expected for the response. In physical units, this can lead to a few nanoseconds for the process.

**Table 1:** Minimal output code (see text) without and with spike latency, respectively **(a, b)**, and with arrival time **(c)** for each input digit.

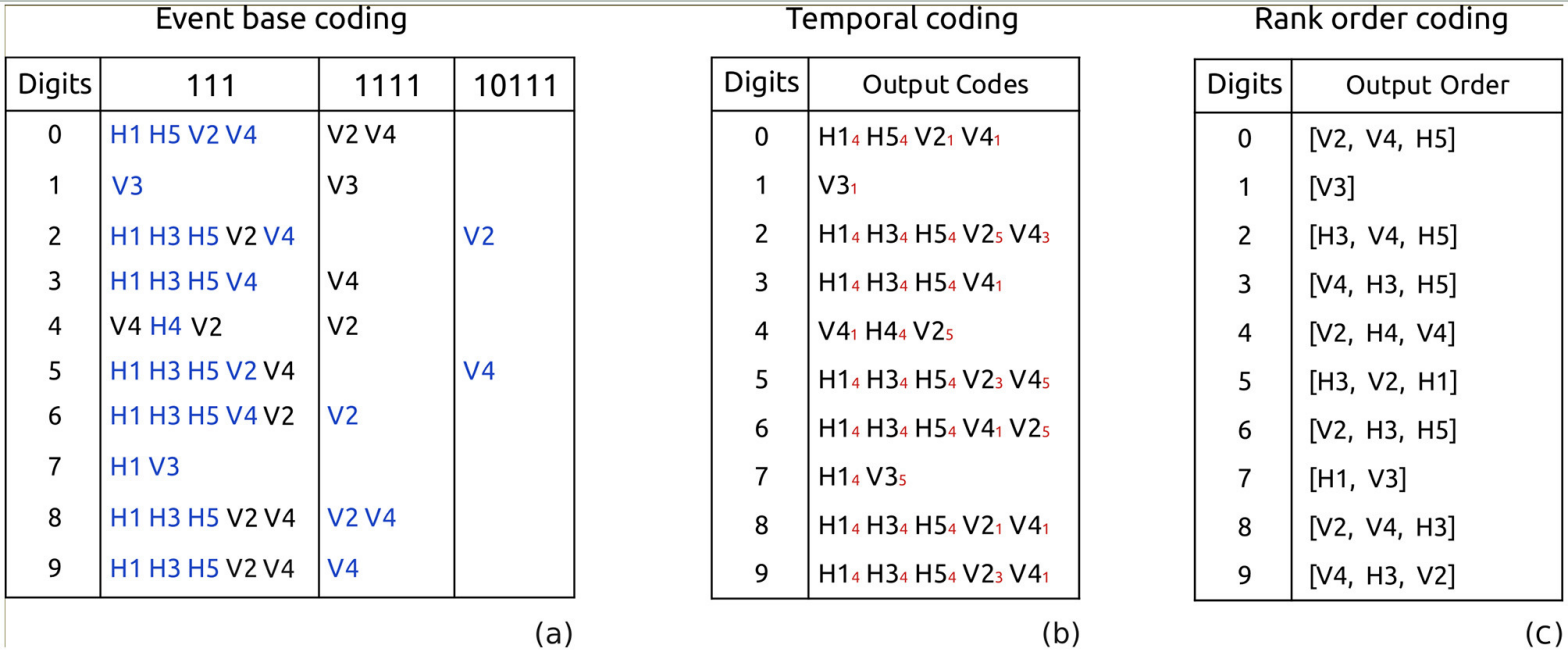

In **(A)**, each column contains the list of receptive fields eliciting a spike for a specific input sequence corresponding to a given digit. The minimum output code is highlighted in blue (the unique code with minimum number of receptive fields). Notice that some receptive fields will be activated with more than one input pattern. In **(B)**, the output code for each digit contains the list of receptive fields eliciting a spike with its arrival rank, from 1 to 5, to each spike based on the number of perturbations and position in the image. In **(C)**, the output code for rank order classification based on the first three arrived spikes, where the lowest latency is first in the list.

#### 3.4.2. Spike-time coding

**Figure 3:**
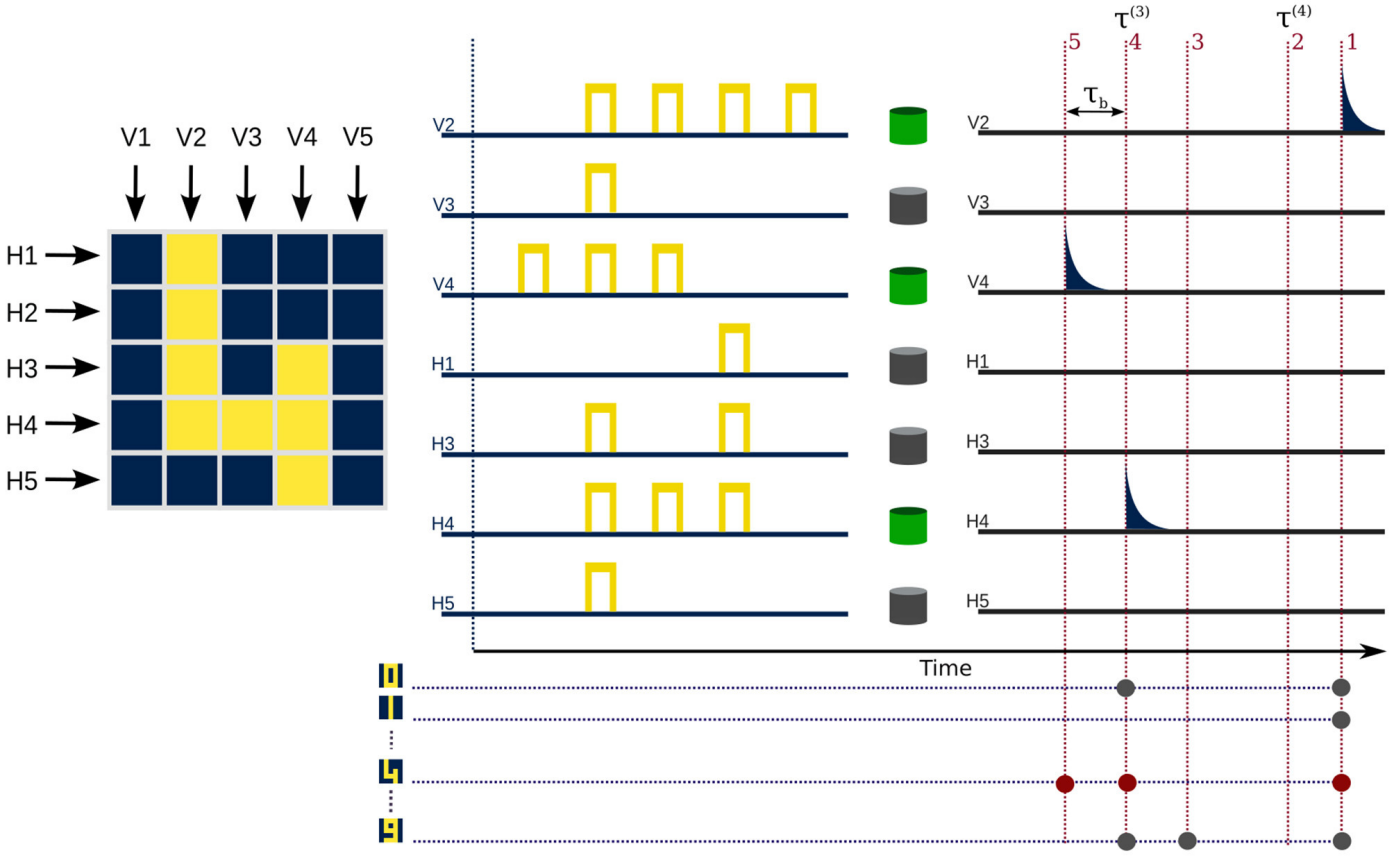
Spike time based coding. Input sequences in chosen receptive fields are sent to micropillar lasers tuned to spike in response to at least three consecutive “1” bits. τ^(3)^ and τ^(4)^ are the latency times for input patterns “111” and “1111,” respectively. Possible spike timings are separated by τ_*b*_ (temporal length of the pixel) and are indicated by labels 1–5. The correct output sequence can be identified and results in the prediction of a given class.

In this second method, we make use of spike latency, one of the fundamental properties of biological neurons that also exists in our artificial photonic neuron. As shown in Figure 2C, the theoretical spike latency difference (δτ_*l*_) between the two input sequences, namely “111” and “1111,” can approach infinity. This difference in latency derives from the evolution of net-gain [*R*(*t*)] and laser intensity [*I*(*t*)] in the different input sequences. To surmount this, we consider the temporal position of the resulting spikes. As illustrated in Figure 3, by choosing a parameter region (μ_0_, *c*, τ_*b*_, and τ_*p*_), where the time latency difference $\delta \tau_{l}=\tau^{\left(3\right)}-\tau^{\left(4\right)}$ is greater than τ_*b*_, we can introduce a coding scheme based on spike time. This spike-time coding scheme, which is shown in Table 1b, assigns a temporal position stamp *k* to each receptive field (*Hn*_*k*_/*Vn*_*k*_), *k* ranging from 1 to 5, fastest to arrive (lowest latency) to slowest, respectively. This leads to a unique code for each digit class.

This method of coding removes the need for the redundant check of receptive fields {*V*2, *V*3, *V*4}, for presence of different patterns, where the temporal position of the output spike (*k*) contains the information of type and location of the pattern all at once. This reduces to seven the number of artificial neurons needed for this coding, while increasing the collected features to 12. By choosing μ_0_ = 2.65 and *c* = 0.5 we can be assured of a delay difference $\delta \tau_{l}\approx 10^{3}$, which is an order of magnitude greater than τ_*b*_ = 50, therefore satisfying the criteria of no overlap of temporal position stamps, regardless of input pattern disposition in receptive fields.

As shown in Figure 3, the output spike time of each receptive field, can be utilized in different ways to decode. The simplest way to realize the decoding scheme consists in the use of a set of ten micropillars, each tuned to respond to a certain pattern of output codes. The system still takes advantage of parallel processing, and therefore, keeps the feature extraction and prediction time to a few nanoseconds.

#### 3.4.3. Rank coding

We can extend the framework of spike-time coding to align with the principles of rank order coding (Thorpe and Gautrais, 1998), where only the order of arrival of spike times is taken into account. To ensure that each the spike resulting from a receptive field is unique and remove accidental degeneracies of spike-times in different receptive fields, we assign a random delay τ_*d*_∈[1, 100] to each receptive field. The range of τ_*d*_ was decided in relation to the delays of other patterns so that it can alter the arrival time effectively. Here we chose τ_*b*_ = [24, 47, 20, 60, 91, 9, 67] for receptive fields {*V*2, *V*3, *V*4, *H*1, *H*3, *H*4, *H*5}, respectively. We use the same number of receptive fields, i.e., 7. We use the parameters μ_0_ = 2.6, *c* = 0.7, and keep τ_*b*_ = 50, such as to have a clear distinction between the three patterns : “111,” “1111,” and “11111.”

As illustrated in Table 1c, by considering only the arrival order of the first three spikes, we can accurately predict the class to which it belongs. During the learning process, the output connection of each class is made to a particular receptive field that has been chosen and optimized to respond to a specific spike arrival order. In the case of digits with fewer than three output spikes, such as 1 and 7, the order is still unique and distinguishable from other classes. Using a more comprehensive dataset would result in a wider range of spike combinations.

It is worth noting that the random delay can be easily implemented by, e.g., adding a small random difference in the pump parameter μ_0_ of each neuron, or could even be “naturally” implemented by the inherent fabrication inhomogeneities of the different microlasers considered. All the random choices are not equivalent and may not lead to an efficient classification. They can thus be considered as hyperparameters for our problem. However, our simulations show that it is rather easy to select a set of random delays suitable for a given task.

## 4. Discussion and conclusion

In this numerical study, we demonstrate the efficacy of incorporating a nonlinear element, specifically laser micropillars, into the classification task. The incorporation of this fundamental nonlinearity serves to elevate the feature space, thereby enabling the utilization of fewer features for the classification task. By using the concept of a receptive field, we can significantly reduce the number of spikes from one spike per one bit, to one spike per multiple bits. This shows how more complex neuron models can be used to advantage to improve the energy consumption of a spiking neural network.

In the event-based method, we observe that utilizing 10 artificial neurons (laser micropillar neurons)—as opposed to the 25 features (of which 15 are distincts) in the original feature space consisting of 5 × 5 images—enabled successful classification of the entire dataset. The classification is based on choosing the right parameters for the micropillar laser, enabling the separation of different input sequences. We find that it can be rather easy to obtain a correct classification requiring a resolution of 10 percent in both μ_0_ and *C*.

We also implemented methods using time coding, which brings the functionality of our system closer to that of biological systems. In the spike-time based and rank based methods, the number of artificial neurons needed is further reduced to 7, representing an absolute minimum for the dataset in question. The reduction in the number of features and subsequently, the number of neurons required, is of significant importance as it allows for offline or extra computation to be avoided, thereby enabling the system to naturally identify distinguishable features and minimized the time and energy required for a classification. The parameters range required in this case is slightly smaller than the one identified in Figure 2A, as can be seen in Figure 2C and still represents a sizable range. Moreover, the rank order classification algorithm is particularly appealing because it only relies on the spike arrival order. This is unlike spike-time based methods, where spike arrival times must be compared to a reference. The classification is effective within the time needed to receive the third fastest spike, which can be on the order of only a few nanoseconds in physical systems (Selmi et al., 2016).

In light of the findings of this theoretical study, the implementation in an experimental setup would serve as a clear demonstration of the computational and learning capabilities of a neuromorphic system based on micropillars. As depicted in Figure 3, this implementation can be achieved using multiple micropillars or by dividing the task into a manageable process for a single pillar through adjustments of the necessary parameters. Using semiconductor sources to pump the micropillars optically and multiple optical modulators to produce optical pulses as input data into the pillars are experimentally feasible approaches for this implementation. Electrical biasing of the micropillar lasers is also a viable and promising technique. We also note that the algorithms presented here are not restricted to these specific setups and could be used in the experimental platforms of Ma et al. (2017) and Skalli et al. (2022).

Scaling up of the network much further or generalization to larger datasets is an open question. However, we would like to stress that our system in its current configuration, in addition to being used as the output of a physical reservoir for a classification task, may also be used prior to a larger classification as an ultra-fast feature selector, in the spirit of convolutional neural networks. It is worth also noting that a multilayer structure can in principle be adopted in this approach since the timing information would simply flow between the layers. In the case of the rank order code, it occurs at the network's final stage and it is no necessary to determine the timing at any other point in the network.

Furthermore, the biological relevance of the temporal models introduced in this study allows for the exploration of more complex, real-world datasets as was used in Van Rullen et al. (1998) for facial recognition using one spike per neuron. Such an algorithm relies on detecting basic facial features and their relative positions which could be performed in hardware with our ultra fast system. The fundamental principle remains the same, identifying the dominant features that define the dataset and allowing the micropillar to reduce the feature space through its nonlinearity. The ranking of the output is also a crucial factor in reducing the time and energy required for more complex tasks where, only specific connections are capable of extracting the necessary information for classification. The potential applications are diverse and, owing to the rapid response time of our artificial neurons, highly convenient. In addition, we point out that the ingredients used by our algorithms stem from very general properties found in many other excitable systems, namely temporal summation and spike latency. Thus, we expect that the algorithms introduced here can be implemented in many other systems.

## Data availability statement

The raw data supporting the conclusions of this article will be made available by the authors on reasonable request.

## Author contributions

AM performed the numerical simulations. AM, LC, and SB analyzed the results and wrote the article. All authors contributed to the development of the ideas and results presented in this work and approved the submitted version of the manuscript.
